# Impact of untreated preoperative asymptomatic bacteriuria in patients undergoing holmium laser enucleation of prostate

**DOI:** 10.1308/rcsann.2024.0027

**Published:** 2024-05-24

**Authors:** D Bheenick, M Conroy, J Bondad, D Dawam, T Young, P Acher

**Affiliations:** Mid and South Essex NHS Foundation Trust, UK

**Keywords:** Urosepsis, HoLEP, Preoperative asymptomatic bacteriuria

## Abstract

**Introduction:**

Treatment of preoperative asymptomatic bacteriuria (ASB) before endoscopic surgery is recommended by European Association of Urology (EAU) guidelines. United Kingdom (UK) practice varies, however, owing to the historical nature of the evidence behind the guidelines, risk of increased antimicrobial resistance, the paradoxical view that treatment of ASB leads to increased infection and inefficiencies in rescheduling. We do not routinely treat ASB in our practice before holmium enucleation of the prostate (HoLEP). To determine the safety of this, we examined our experience focusing on the infective complications.

**Methods:**

Retrospective data collection was performed on consecutive patients undergoing HoLEP between 2015 and 2020. Indication, preoperative urine cultures and infective complications were recorded. No patients were pretreated with oral antibiotics. All patients received intravenous antibiotics on induction and routine postoperative oral antibiotics at the surgeon's discretion.

**Results:**

Some 443 patients were studied. No urosepsis occurred in the 125 patients with ASB compared with 2 of 318 patients (0.6%) with no growth on preoperative urine culture. Twenty-nine (7%) patients were treated with oral antibiotics for symptomatic postoperative complications (urinary tract infection without fever, epididymitis and haematuria). ASB did not predict for infective complications (urosepsis odds ratio [OR]: 0.50 *p*=0.66; oral antibiotics OR: 0.97 *p*=0.93).

**Conclusion:**

Not treating ASB before a HoLEP procedure is safe. This supports the judicious use of antimicrobials preoperatively. Other modalities of endoscopic surgery should be similarly assessed.

## Introduction

Asymptomatic bacteriuria (ASB) has led to debate between clinicians within urology. Although some advocate treating ASB before any urological interventions, others are questioning this routine practice, calling for closer investigations and a more robust evidence base.

The term ASB refers to the isolation of bacterial species in significant counts (≥10^5^cfu/ml) from a clean catch urine specimen of a patient without any urinary symptoms or acute signs.^1^ Since the discovery of ASB, its clinical significance and management has been uncertain. Published medical literature and guidelines (European Association of Urology [EAU] 2022) focus on positive interventions especially before endourological procedures such as prostate surgery.^2,3^ However, most of the evidence used to support the guidelines dates to the 1980s, before the routine use of antibiotics on induction. No guidelines clearly define the timing or duration of treatment for ASB. Some studies suggested starting treatment 72h before the intervention although treating ASB does not prevent patients from being recolonised.^4,5^ Such early treatment may, therefore, seem excessive and allow the opportunity for further infection to develop before the procedure with increased antimicrobial resistance.

ASB is common in the general population among adults. Prevalence of ASB varies with age, gender and sexual activity. In elderly men, the prevalence of ASB is reported to be between 4% and 7%.^6^ Major contributing factors include prostatic enlargement leading to bladder outlet obstruction, resulting in incomplete bladder emptying and urinary stasis.^7^

Many patients with bladder outlet obstruction are managed with an indwelling catheter for adequate bladder drainage while awaiting their surgical intervention. Inevitably patients with long-term indwelling catheters will have ASB, secondary to colonisation of the catheter.^8^ Multiple studies have emphasised that the longer the duration of catheterisation, the higher the risk of developing catheter-associated bacteriuria. The incidence of bacteriuria in patients with an indwelling catheter is 3–7% per day.^9^ After chronic catheterisation (defined as 28 days or more), almost 100% (98.4%) of patients will have bacteriuria and 77% would have polymicrobial bacteriuria.^10^

The EAU guidelines (2022) on urological infections suggest that before endourological interventions on the urinary tract, especially where there is breach of the mucosa, a urine culture should be taken, and in the case of ASB preoperative treatment is advised.^2^ Several randomised controlled trials have compared the effect of antibiotic treatment with no treatment in urological procedures such as transurethral resection of the prostate (TURP) and of bladder tumour (TURBT). Patients given antibiotics before surgery had reduced postoperative complications, such as fever, symptomatic urinary tract infection (UTI) and septicaemia. However, the studies quoted in the guidelines were all performed in the 1980s and compare patients who did not receive any antibiotics at all perioperatively.^11–14^

Contemporary practice is to administer periprocedural antibiotic prophylaxis in major urological surgeries because it has been shown to reduce postoperative infectious complications.^2,9^ However, very little research has been conducted regarding whether periprocedural prophylactic antibiotics are effective in reducing postinfective complications in patients who did not receive preoperative treatment for ASB.

**Table 1:** Indications and infective complications

Indication	Total *n*	Asymptomatic bacteriuria			No growth on culture		
		Total *n*	Urosepsis *n*	Complication treated withoral antibiotics *n* (%)	Total *n*	Urosepsis *n* (%)	Complication treated withoral antibiotics *n* (%)
Urinary retention	195	105	0	7 (7)	90	1 (1)	8 (9)
LUTS	248	20	0	1 (5)	228	1 (0.4)	13 (6)
Total	443	125	0	8 (6.4)	318	2 (0.6)	20 (7)

LUTS = lower urinary tract symptoms

This raises the question – should patients with ASB undergoing endourological procedures receive treatment preoperatively, or is perioperative antimicrobial prophylaxis adequate?

It is our practice not to treat ASB preoperatively with antibiotics. This retrospective study was therefore designed to determine if patients with untreated ASB before undergoing holmium laser enucleation of prostate (HoLEP) had a higher risk of developing postoperative infective complications compared with patients with no bacterial growth in the urine before surgery.

## Methods

A retrospective study was conducted between 2015 and 2020 at Southend University Hospital (now part of Mid and South Essex NHS Trust), UK. This was approved as an audit by the hospital governance committee.

Some 447 consecutive patients undergoing HoLEP were selected. Four patients with ASB who received antimicrobial therapy before surgery were excluded.

Data collection was performed by using the hospital database. Full clinical records were reviewed. The information was categorised into indication of the procedure including lower urinary tract symptoms (LUTs) or urinary retention (patients with indwelling catheters), preoperative urine cultures indicating ASB or no bacterial growth and infective postoperative complications. Complications were further subcategorised into patients being admitted with urosepsis or those requiring only oral antibiotics for UTI without fever, epididymo-orchitis, haematuria and prostatitis. By using an integrated system with the primary care records available, patients who developed postinfective complications treated with oral antibiotics in the community were also identified.

All patients received intravenous antibiotics at induction of anaesthesia guided by urine culture sensitivities or as per the local trust protocols. Local trust protocol was gentamicin (2mg/kg); co-amoxiclav was added for those patients with an indwelling catheter at the time of surgery. Postoperative oral antibiotics (3 days of trimethoprim, or co-amoxiclav if trimethoprim contraindicated) were prescribed as per the operating surgeon’s discretion to a small number within both groups of patients.

## Results

**Figure 1:**
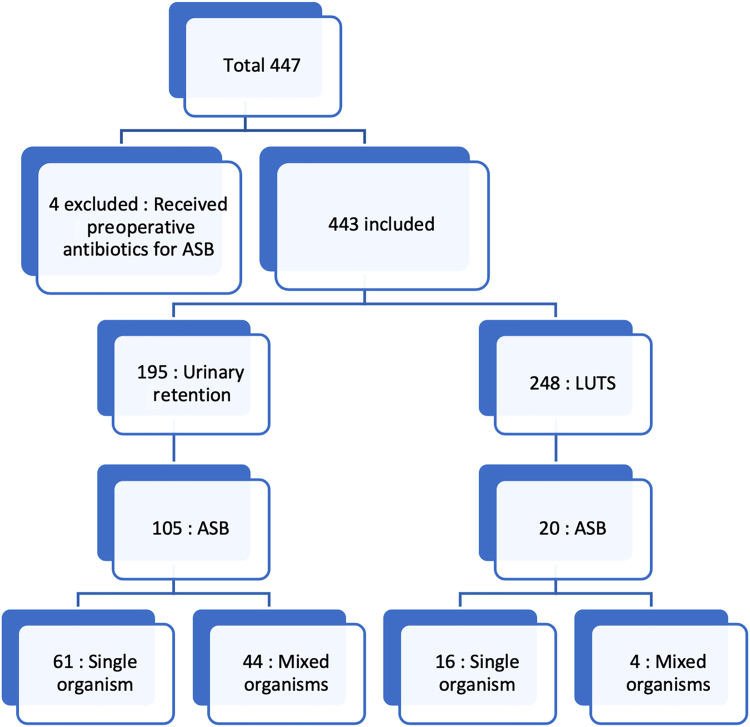
Classification of patient under study. ASB = asymptomatic bacteriuria; LUTS = lower urinarty tract symptoms

In total 447 patients undergoing HoLEP were identified, out of whom four were excluded because they were prescribed oral antibiotics preoperatively by other clinicians. Indications for HoLEP are outlined in Table 1. A total of 443 patients were studied (Figure 1). There was no significant difference in spread of relevant comorbidities (e.g. diabetes) between the two groups.

Some 125 patients were found to have ASB before surgery. Those with an indwelling urethral catheter (urinary retention) had a higher percentage of ASB at 84% (105 of 125) compared with those without a catheter (LUTS) at 16% (20 of 125) (Figure 1).

In patients with an indwelling urethral catheter and ASB, 61 grew a single organism and 44 had mixed growth. In the LUTS group (patients with no catheter and ASB), 16 had a single organism in their urine specimen and 4 had a mixed growth.

No urosepsis occurred in the ASB group compared with 2 in the remaining 318 patients (0.6%) with no growth on preoperative urine culture. Of the two patients who developed urosepsis, one had an indwelling urethral catheter for urinary retention before surgery. Both patients were admitted and treated with parenteral antimicrobial therapy with good recovery (Table 2).

**Table 2:** Urine culture in patients with postoperative urosepsis

Preoperative urine culture	Catheter	Postoperative urine culture
No growth	Yes	Pseudomonas species
No growth	No	Coliform species

A total of 29 (7%) patients of the whole cohort were treated with oral antibiotics for symptomatic postoperative complications: 8 (6.4%) in those with ASB and 21 (7%) in patients with no bacterial growth on urine culture preoperatively.

The most common condition, for which 24 of the 29 patients were given oral antibiotics, was suspected UTI. However, only 11 of 24 (37.9% of the 29 patients treated with oral antibiotics) patients had a positive urine culture and the 2 patients admitted with visible haematuria and suspected UTI had no growth on their subsequent urine cultures. Three (10.3%) patients had clinically diagnosed epididymo-orchitis and one patient was treated for prostatitis. All patients were treated with oral antibiotics with no further complications. Patients with ASB requiring postoperative antibiotics did not grow similar bacteria on the postoperative urine culture (Table 3).

**Table 3:** Information comparing preoperative and postoperative urine cultures in patients who developed infective complications

Postoperative complication	Urinary retention			Lower urinary tract symptoms		
	Urine culture preoperatively	Urine culture postoperatively	Number	Urine culture preoperatively	Urine culture postoperatively	Number
Urosepsis	No growth	*Pseudomonas* species	1	No growth	Mixed growth	1
Suspected/confirmed UTI	Mixed growth	*Enterococcus*	1	*Citrobacter*	*Escherichia coli*	1
		*Escherichia coli*	1			
		*Pseudomonas*	1			
	Coliform species	Heavy mixed growth including *Candida* species	1			
		No growth	1			
	*Enterococcus*	No growth	1			
	No growth	*Enterococcus*	1	No growth	*Enterococcus*	1
		Coliform species	1		Coliform species	1
		*Pseudomonas*	1		*Klebsiella oxytoca*	1
		No growth	3		No growth	7
Epidymo-orchitis	No growth	*Enterococcus*	1	No growth	*Enterococcus*	1
					*Pseudomonas*	1
Haematuria	Mixed growth	No growth	1			
	No growth	No growth	1			
Prostatitis				No growth	No urine test	1

UTI = urinary tract infection

In the 125 patients with ASB, 7 in 105 (7%) in the catheterised group required oral antibiotics for symptomatic infections after surgery compared with 1 in 20 (5%) patients in the LUTS category.

For patients with no preoperative bacterial growth on urine culture, 8 (9%) with urinary retention and 13 (6%) with LUTS developed infective complications.

As shown by the results, ASB did not predict for postoperative infective complications (urosepsis odds ratio [OR]: 0.50 *p*=0.66; oral antibiotics OR: 0.97 *p*=0.93).

## Discussion

In this retrospective study, preoperative ASB was not associated with higher rates of postoperative urosepsis or positive urine culture results among symptomatic patients undergoing HoLEP. A similar percentage of patients in both cohorts developed infective complications, such as UTI. These data therefore seem to suggest that there is no benefit in reducing postoperative infective complications in treating ASB before HoLEP.

The EAU 2022 guidelines on urological infections advise treatment of ASB preoperatively, highlighting four studies as the evidence base. Table 4 gives a summary of the studies quoted.^11–14^

**Table 4:** Summary of studies quoted in the European Association of Urology guidelines (2022)

Study	Year	Sample size	Procedure	Preoperative antibiotics	Control	Results
Prospective RCT	1984	192	TURP	98	94	Frequency of bacteriuria
						• Cefotaxime group: 43% preoperatively and 18% at 6 weeks postoperatively. • Control group: 40% preoperatively and 42% at 6 weeks postoperatively. Postoperative complication
						• Cefotaxime group: 0 • Control group: 1 septicaemia, 7 upper UTI (*p*<0.01)
Prospective RCT	1987	176	TURP	Group 1: Short course ciprofloxacin Group 2: Prolonged course of ciprofloxacin	—	Bacteriuria postoperatively Group 1: 35% positive Group 2: 10% positive Control group: 82% Reduced frequency of postoperative bacteriuria (*p*<0.01) and severe infectious complications (*p*=0.004) in treatment group
Prospective nonrandomised study	1982	565	Transurethral operation (TURP, urethral dilation, TURBT)	180 (positive urine culture)	179 (positive urine culture)	NB: 206 with sterile urine were also included Cases of septicaemia • Control group: 11 cases, including 1 death (6.15%) • Antibiotic group: 0 • Sterile urine group: 3
Prospective nonrandomised study	1984	112	TURP and other transurethral procedures	63	25	Percentage of positive blood culture taken intraoperatively
						• Control group: 60%
						• Antibiotic group (received 24h before surgery): 21%
						• Antibiotic group received >24h before surgery: 0
						No septicaemia in whole cohort

RCT = randomised controlled trial; TURBT = transurethral resection of bladder tumour; TURP = transurethral resection of the prostate; UTI = urinary tract infection

This study is the first to question the recommendations of treating patients with ASB before HoLEP and highlight that, in our cohort, ASB did not affect postoperative infection rates.

In this study, all patients undergoing HoLEP received intravenous perioperative antibiotics at induction of anaesthesia. As stated by the EAU and American Urological Association guidelines for urological infections, there is a high level of evidence that single-dose prophylactic antibiotics reduce the rate of infectious postoperative complications in patients undergoing TURP.^2,3^ As demonstrated in this study, perioperative prophylaxis with single-dose antibiotics is just as effective for patients with ASB in reducing postoperative complications. Therefore, pretreating patients with ASB before HoLEP surgery does not seem to be of benefit from an infective outcomes perspective. The further burden placed on health services with having to treat ASB patients and re-culture urine is also a significant factor to consider.

Screening patients for bacteriuria before diagnostic and therapeutic procedures would, however, still be important because it would guide antimicrobial coverage in conjunction with the procedure. The choice of antibiotics would obviously depend on local pathogen prevalence and type of procedure, but a urine culture would help identify multiresistant organisms.^2^

This study has raised an important point in the management of ASB in patients undergoing HoLEP. Identifying other surgical procedures whereby the postoperative outcome is unchanged by not treating ASB will not only improve cost-effectiveness, but also result in more judicious use of antimicrobials, meeting the goals of antimicrobial stewardship. As highlighted by the ‘Choosing Wisely’ campaign, not treating ASB in most circumstances is recommended, given concerns about the harms of antimicrobial use, such as emergence of antimicrobial resistance, adverse drug events and increases in *Clostridium difficile* infection because treating ASB is without any demonstrated value.^5^ Moreover, there could be a reduction in on-the-day cancellations from patients found to have ASB.

The EAU guidelines (2022) reference research performed before the existence of HoLEP as a surgical option and focus mainly on TURP, whereas now a multitude of surgical options are available for treating bladder outlet obstruction.^2^ The studies highlighted in these guidelines do have a number of limitations. Three of the studies have poor sample sizes, with fewer than 192 participants (112–192) across both randomisation arms. Cafferkey *et al* do have a larger sample size (565), but 206 of these patients had sterile urine preoperatively and therefore part of these data cannot be reliably applied to the ASB cohort and may have skewed the presented results.^13^ Whether the procedure performed transurethrally leads to a difference in infective outcome rate is an area not fully researched and limited robust evidence exists regarding this. Although, given that some bladder outlet obstruction surgeries are now becoming more ‘minimally invasive’, it could be inferred that these provide lower risk of introducing infective pathogens due to limited breaching of the mucosal barrier.

To our knowledge, this is the first study conducted to assess the impact of untreated preoperative ASB in patients undergoing HoLEP. Although not a randomised prospective study, a large cohort size has been evaluated, reflecting clinical practice over a 5-year period.

### Study limitations

We acknowledge a few limitations with our work. Factors suggested to have an impact on postoperative complication rate include patient comorbidities, laser time for the HoLEP and duration of catheterisation after surgery. These data are not accurately recorded for our cohort and so formal analysis of the impact of this could be an area for further prospective research. Some patients received a short (5-day) course of antibiotics after the procedure, no standardised criteria were followed as to which patients received antibiotics after the procedure because this was based on clinician preference.

Only patients undergoing HoLEP were included in this study. Further prospective studies should be conducted to assess whether similar findings will be found in patients undergoing other bladder outlet obstruction procedures such as TURP. Our findings raise important issues regarding antimicrobial use and stewardship, and highlight a gap in current, up-to-date knowledge and evidence for clinical practice.

## Conclusion

The presence of ASB in patients undergoing HoLEP does not require preoperative antibiotics. However, it is important that clinicians give adequate perioperative antibiotics guided by local policy and urine culture sensitivities. Judicious use of antibiotics is advised, especially considering increasing prevalence of antimicrobial resistance. Although current guidelines advocate treatment of ASB before urological procedures, further research is required to establish whether this is necessary in the current era.
